# The Testosterone Effect on Metabolic and Urologic Outcomes in Patients with Nonfunctioning Pituitary Macroadenomas and Hypogonadotropic Hypogonadism

**DOI:** 10.1155/2019/2356580

**Published:** 2019-11-03

**Authors:** Guadalupe Vargas-Ortega, Gabriel Pérez-Villarreal, Andrés Ramírez de Santiago, Lourdes Balcázar-Hernández, Victoria Mendoza-Zubieta, Oscar Landa-Gutierrez, Carlos Estrada-Robles, Baldomero González-Virla

**Affiliations:** From the Endocrinology Department, Hospital de Especialidades, Centro Médico Nacional Siglo XXI, Instituto Mexicano del Seguro Social, Mexico City, Mexico

## Abstract

**Objective:**

To evaluate cardiovascular risk, metabolic profile, low urinary tract symptoms (LUTS), and sexual function in patients with nonfunctional pituitary macroadenoma (NFPMA) and hypogonadotropic hypogonadism with testosterone therapy (TTh).

**Methods:**

A retrospective clinical study at a tertiary care center was performed in 101 men with NFPMA, HH, and TTh; metabolic profile, cardiovascular risk, International Prostate Symptoms Score (IPSS), and International Index of Erectile Function 5 (IIEF-5) scores were evaluated before initiation of TTh and at the last checkup with TTh.

**Results:**

Age was 49.3 ± 8.8 years; *T* before TTh was 195 ng/mL (101–259) vs. 574 (423–774) at the last checkup. The time of TTh administration was 34 months (12–72). An increase in triglyceride levels (200 (153–294) vs. 174 (134–233) mg/dL; *p*=0.03), dyslipidemia (40% vs. 52%; *p*=0.03), and MetS (25% vs. 34%; *p*=0.05) was corroborated. A statistical difference in the Globorisk score and cardiovascular (CV) risk stratification was not found. IIEF-5 score was 15.5 ± 6.5 vs. 17.8 ± 5.3 (*p*=0.11). An improvement in penetration quality (2.0 ± 1.5 vs. 2.6 ± 1.3; *p*=0.05), erection after penetration (1.8 ± 1.2 vs. 2.5 ± 1.6; *p*=0.02), completion of intercourse (1.8 ± 1.2 vs. 2.4 ± 1.3; *p*=0.03), and satisfaction of sexual intercourse (1.8 ± 1.3 vs. 2.5 ± 1.5; *p*=0.01) was evidenced. IPSS score was 6 (IQR 2–10) vs. 7 (IQR 4–12); *p*=0.30. A lower rate of intermittency (14% vs. 3%; *p*=0.02), urgency (39% vs. 16%; *p*=0.01), and episodes of nocturia (18% vs. 4%; *p*=0.02) was found. An increase of hematocrit (44.1 ± 4.4 vs. 47.3 ± 4.4%; *p*=0.001), hemoglobin (14.9 ± 1.4 vs. 15.9 ± 1.4 g/dL; *p*=0.001), and prostatic specific antigen (0.59 (0.43–1.19) vs. 0.82 (0.45–1.4) ng/mL; *p*=0.02) was evidenced during TTh.

**Conclusion:**

TTh in young men with NFPMA improves LUTS, sexual function, and some metabolic parameters, and it is relatively safe in the prostatic context.

## 1. Introduction

Hypogonadism in men is a clinical syndrome that results from failure of the testis to produce physiological levels of testosterone and a normal number of spermatozoa due to disruption of one or more levels of the hypothalamic-pituitary-testicular axis [1, 2].

Androgen deficiency (AD) is characterized by chronic fatigue, diminution of libido, depression, erectile dysfunction, anhedonia, abdominal obesity, and diminution on quality of life (QoL). Low testosterone levels are associated with hypertension, hypercholesterolemia, diabetes, osteoporosis, and ischaemic heart disease. Testosterone (*T*) and 5*α*-dihidrotestosterone (5-DHT), its metabolite, regulate the energetic metabolism, muscular growth, and maintenance; inhibit adipogenesis; and modulate male reproductive and sexual function. The role of *T* in the regulation of bone metabolism, erythropoiesis, hair growth, and endothelial and liver functions is well established. AD impacts negatively in male health and is associated with an increase in weight, adiposity, waist circumference, insulin resistance, diabetes, hypertension, inflammation, atherosclerosis, metabolic syndrome, infertility, cardiovascular disease, erectile dysfunction, and mortality. AD is related with decreased lean body mass, low bone density, and anemia [2–5]. There has been an emerging controversy in the past several years regarding the safety of testosterone replacement therapy (TTh) due to a suggested increased risk of cardiovascular disease (CVD) among its users. In 2015, the United States Food and Drug Administration (FDA) stated that TTh is only approved for congenital hypogonadism or acquired damage to the brain, pituitary gland, or testes. TTh has been associated with a positive effect on cardiovascular disease, low urinary tract symptoms (LUTS), and erectile function [1, 2]. Hypogonadrotropic hypogonadism (HH) is common in patients with nonfunctional pituitary macroadenomas (NFPMA); but the specific effect of TTh in NFPMA has been less studied. The aim of this study was to evaluate cardiovascular risk, metabolic profile, low urinary tract symptoms (LUTS), and erectile dysfunction in patients with NFPMA and HH treatment with testosterone.

## 2. Materials and Methods

### 2.1. Patients

A retrospective clinical study was conducted at a tertiary care center. A total of 118 men with NFPMA and hypogonadotropic hypogonadism on TTh, aged less than 65 years, enrolled in the clinic of pituitary adenomas at Hospital de Especialidades, Centro Médico Nacional Siglo XXI, IMSS, in México City, were included. Characteristic of metabolic profile, cardiovascular risk, low urinary tract symptoms (LUTS), and sexual function were evaluated before TTh and at the last checkup. The last checkup was the last medical visit to our clinic during the evolution. All patients were treated with transsphenoidal surgery for NFPMA between 2010 and 2017. Hypogonotropic hypogonadism (HH) was defined as a low *T* concentrations (<300 ng/dL) and low or inappropriately normal gonadotropin [1]. TTh consisted in the intramuscular administration of testosterone enanthate 250 mg every 3 weeks. In the clinic of pituitary adenomas, the protocol for initiation and monitoring of TTh consisted in (1) clinical and biochemical evaluation of patients at baseline, 3–12 months after TTh initiation, and then annually [biochemical evaluation included the serum measurements of glucose, glycosylated hemoglobin (HbA1c), triglycerides, uric acid, total cholesterol (TC), high-density lipoprotein cholesterol (HDL-C), and low-density lipoprotein cholesterol (LDL-C), aspartate aminotransferase (AST), and alanine aminotransferase (ALT)]; (2) monitoring *T* concentrations 3–6 months after initiation of TTh; (3) evaluation of hematocrit, at baseline, 3–6 months after TTh, and then annually (TTh was suspended if hematocrit increased >54%); and (4) prostatic-specific antigen (PSA) and digital rectal examination at baseline, 3–12 months after initiating TTh, and then in accordance with guidelines for prostate cancer screening depending on the age and race of the patient. Urological consultation was obtained if there is an increase in Delta PSA concentration more than 1.4 ng/mL within 12 months of initiating TTh, PSA > 4 ng/mL at any time, and detection of a prostatic abnormality or worsening of LUTS [1]. For TTh initiation, patients with a history of prostate or breast cancer, unevaluated prostate nodule or induration, PSA > 4 ng/mL, PSA > 3 ng/mL with first-degree relatives with prostate cancer, hematocrit more than 50%, severe LUTS associated with benign prostatic hypertrophy indicated by IPSS > 19, and uncontrolled or poorly controlled congestive heart failure were excluded. Delta of PSA was the difference between PSA at 12 months after TTh and PSA before treatment [1].

Also, we evaluated the glucose, HbA1c, triglycerides, uric acid, TC, HDL-C, LDL-C, AST, ALT, total testosterone, hemoglobin, hematocrit, and PSA levels measured before TTh and at the last checkup.

LUTS describes the symptoms related to alterations of the lower urinary tract (bladder, prostate, and urethra). The International Prostate Symptoms Score (IPSS) was used to measure the intensity of LUTS. IPSS had 8 items, seven related with LUTS (a total score of 35) and one related with QoL (a total score of 6). IPSS > 19 indicated high intensity of symptoms [1]. Erectile functions domains (orgasmic function, sexual desire, intercourse satisfaction, and overall satisfaction) were assessed with the International Index of Erectile Function (IIEF-5). IIEF-5 and IPSS were performed before TTh and at the last checkup [2].

We evaluated the presence of metabolic syndrome (MetS) and the Globorisk score before TTh and at the last checkup. MetS was defined according to the National Cholesterol Education Program (NCEP) expert panel on detection, evaluation, and treatment of high blood cholesterol in adults (Adult Treatment Panel III); MetS was diagnosed with three or more of the following: waist circumference greater than 102 cm, blood pressure higher than 130/85 mmHg or antihypertensive drug medication, fasting triglycerides higher than 150 mg/dL, HDL-C less than 40 mg/dL or a previously treated dyslipidemia, and fasting glucose over 100 mg/dL or diabetes treatment. Patients with diabetes had treatment with metformin and/or glyburide as oral antidiabetic drugs or insulin (glargine or NPH insulin). Patients with hypertension received enalapril, losartan, or amlodipine. Men with dyslipidemia received atorvastatin and/or bezafibrate. Globorisk score was calculated for predicting the cardiovascular risk of fatal cardiovascular disease (CVD) before TTh and at the last checkup. Globorisk score is a prediction equation of 10-year risk of fatal CVD standardized and recommended in our population. A score <3% predicted a low CVD risk, 3–10% moderate CVD risk, and ≥10% a high fatal CVD risk [6].

For hypopituitarism approach, we followed the method of our series in Vargas-Ortega et al. [4]. Hypocortisolism was defined as morning cortisol levels below 3 *μ*g/dL; central hypothyroidism was defined as free thyroxine (FT4) levels lower than 0.5 ng/dL, regardless of thyroid-stimulating hormone (TSH) concentration. Hyposomatotropism was defined as the insulin growth factor (IGF-1) level below the lower limit of normality established for age and gender. Panhypopituitarism was diagnosed if three or more pituitary-hormonal axes were affected. All patients with hypopituitarism received hormone replacement therapy with levothyroxine and prednisone. The replacement dose of daily levothyroxine was calculated at 1.6 mcg/kg body weight per day. Prednisone was indicated at a dose of 5 mg orally once daily (with adjustments of doses during illness, surgery, or another stress situation). Patients with hyposomatotropism did not receive growth hormone replacement because of economic limitations. At the last checkup, the adequate hormonal replacement was determined when free thyroxine (FT4) levels were at a normal range according to the assay method and testosterone levels higher than 300 ng/dL in men [4]. The present study protocol was reviewed and approved by the Institutional Review Board of Hospital de Especialidades Centro Médico Nacional Siglo XXI, IMSS (approval No. R-2017-3601-216). All subjects had informed consent when they were enrolled.

### 2.2. Biochemical Analysis

For biochemical determinations, 8 mL of blood was collected in BD Vacutainer tubes (BD Franklin Lakes, New Jersey, EEUU) and was centrifuged at 3150 ×*g* for 15 minutes in an Allegra X-22 centrifuge (Beckman Coulter Inc., EEUU) to obtain the serum, which was analyzed with a glucose measuring kit, TC, HDL-C, triglycerides, and uric acid, from the COBAS brand (2010 Roche Diagnostics, Indianapolis, EEUU) with a photocolorimetry technique by a spectrophotometer Roche Modular P800 (2010 Roche Diagnostics, Indianapolis, EEUU). For the HDL-C determination, the samples were treated with modified enzymes by polyethylene glycol and dextran sulfate and were analyzed with the same technique. The determination of glycated hemoglobin (HbA1c) was made by immunoanalysis with turbidimetric inhibition, using the COBAS brand kit (2010 Roche Diagnostics, Indianapolis, EEUU) with an intra- and interassay coefficient of variation (CV) of 1.6 and 3.5%. The LDL-C levels were obtained by the Friedewald formula: LDL-C mg/dL = CT mg/dL − HDL-C mg/dL + triglycerides mg/dL/5, as long as the triglyceride concentration was not greater than 400 mg/dL. Alanine transaminase (ALT) (intra- and interassay CV of 3.1 and 9.9%) and aspartate transaminase (AST) (intra- and interassay CV of 2.7 and 2.9%) were measured for transaminases; hemoglobin and hematocrit kits were measured from the COBAS brand (2010 Roche Diagnostics, Indianapolis, EEUU) with a photocolorimetry technique by a spectrophotometer Roche Modular P800 (2010 Roche Diagnostics, Indianapolis, EEUU). Testosterone (intra- and interassay CV < 20%), LH (intra- and interassay CV of 2.7 and 3.1%), FSH (intra- and interassay CV of 3.6 and 4.5%), and prostate-specific antigen (intra- and interassay CV of 3.4 and 6.5%) were measured by electrochemiluminescence immunoassay (COBAS, Roche, EEUU).

### 2.3. Statistical Analysis

Quantitative variables were described as means ± standard deviation (SD) or medians and interquartile ranges (IQR) according to their distribution, which was ascertained by means of the Shapiro-Wilks test. Proportions were used for qualitative variables (expected frequency and prevalence). To establish associations between continuous variables, we used Wilcoxon, and, for qualitative variables, chi square test or Fisher's, according to the expected value in the boxes was used. We considered a *p* value of < 0.05 as significant. We used SPSS 22 and STATA 11 as statistical software.

## 3. Results

### 3.1. Clinical Characteristics

A total of 118 patients with NFPMA and HH on TTh were included; the mean age was 49.3 ± 8.8 years; median of *T* before TTh was 195 ng/mL (IQR, 101–259) vs. 574 (423–774) at the last checkup. The follow-up duration of TTh was 34 months (IQR, 12–72). The most common clinical findings were bitemporal hemianopia (68%) and headache (71%); 5% (*n*=6) had oculomotor nerve palsy; and 7% (*n*=8) had intracranial hypertension. In tumor characteristics, cephalocaudal diameter was 34.2 ±11.4 mm, transverse 28.1 ± 8.2, and anteroposterior 29.5± 7.5, with a tumor volume of 23800 (RIC, 18745–41664)mm^3^; 44% (*n*=52) was giant pituitary adenomas; and 16% (*n*=19) was invasive; the 59% (*n*=70) of patients had hypothyroidism, 35% (*n*=41) had hypocortisolism, and 9% (*n*=11) had hyposomatotropism.

### 3.2. Anthropometric and Metabolic Characteristics

Before TTh, weight was 79 ± 14 kg and BMI 27.6 kg/m^2^ (IQR 26.4–28.7) vs. weight of 80.7 ± 13.6 kg and BMI 28.8 (IQR 25.4–31.6) kg/m^2^ at the last checkup. The clinic and biochemical characteristics of metabolic profile before TTh and at the last checkup are reported in Table 1; only triglycerides had statistical difference (*p*=0.03). A change in the rate of dyslipidemia (40% vs. 52%; *p*=0.03) and MetS (25% vs. 34%; *p*=0.05) was evidenced. There were no differences between the frequencies of obesity (30% vs. 32%; *p*=0.40), arterial hypertension (22% vs. 27%; *p*=0.85), and type 2 diabetes (17% vs. 19%; *p*=0.84).

### 3.3. Globorisk Score

The Globorisk score before TTh was 7 (4–10) vs. 7 (5–11) at the last checkup, without statistical difference. There were no changes in cardiovascular risk stratification before TTh vs at the last checkup: low CVD risk 56% vs 46% (*p*=0.10), moderate CVD risk 5% vs. 8% (*p*=0.08), and high CVD 0% vs. 4% (*p*=0.06). The 18% of patients had antihypertensive treatment, 20% statins, and 21% fibrates at the last checkup.

### 3.4. International Index of Erectile Function (IIEF-5)

IIEF-5 score was 15.5 ± 6.5 before TTh vs. 17.8 ± 5.3 at the last checkup (*p*=0.11). Patients referred a better penetration quality (2.0 ± 1.5 vs. 2.6 ± 1.3; *p*=0.05), erection after penetration (1.8 ± 1.2 vs. 2.5 ± 1.6; *p*=0.02), completion of intercourse (1.8 ± 1.2 vs. 2.4 ± 1.3; *p*=0.03), and satisfaction of sexual intercourse (1.8 ± 1.3 vs. 2.5 ± 1.5; *p*=0.01) (Table 2).

### 3.5. International Prostate Symptoms Score (IPSS)

The IPSS score was of 6 points before TTh (IQR 2–10) vs. 7 (IQR 4–12) at the last checkup (*p*=0.30). All patients had less than 19 points, which placed them in a mild/moderate intensity of LUTS. When we independently compared each item of IPSS, there were no differences in urinary frequency, incomplete bladder emptying, or straining.

We evidenced a lower rate of intermittency (14% vs. 3%, *p*=0.02), urgency (39% vs. 16%, *p*=0.01), and episodes of nocturia (18% vs. 4%, *p*=0.02) at the last checkup. There were no changes in QoL evaluation (1.92 ± 1.27 vs. 1.87 ± 1.35; *p*=0.70) (Table 3).

### 3.6. Adverse Effect of Testosterone Therapy

During the monitoring of TTh, we evidenced a significant increase of hematocrit (44.1 ± 4.4% vs. 47.3 ± 4.4%, *p*=0.001) and hemoglobin (14.9 ± 1.4 g/dL vs. 15.9 ± 1.4 g/dL, *p*=0.001); any patient had clinical repercussions or indications for a specific treatment. An increase of aspartate aminotransferase (AST) was evidenced; however, its values remained in the normal range.

PSA had a significant increase (0.59 ng/mL (0.43–1.19) vs. 0.82 (0.45–1.4); *p*=0.02); four patients had a delta of *T* > 1.4 ng/dL at the last checkup, requiring withdrawing of TTh and an integral urological evaluation. One patient had acinar adenocarcinoma of prostate, with Gleason pattern of 6 (3 + 3), grade 1, without linfovascular invasion but perineural invasion. Transurethral resection of prostate was performed and currently the patient has not evidenced cancer persistence or recurrence (Table 4). The statistical power of our study to cardiovascular outcomes was of 0.34 vs. 0.96 for urologic outcomes.

## 4. Discussion

The frequency of HH in nonfunctional pituitary adenoma varies from 30 to 80% and is associated with diminution of QoL, depression, sexual dysfunction, depressed mood, decreased motivation, fatigue, mild cognitive impairment, osteoporosis, cardiovascular disease, increased body weight, adiposity, waist circumference, insulin resistance, type 2 diabetes, hypertension, inflammation, atherosclerosis, cardiovascular disease, and mortality [2, 3].

The information about CVD risk and metabolic profile in patients with NFPMA has been studied. Recently, we evidenced that NFPMA patients have a significant increase risk for hypercholesterolemia (SMR 1.68, 95% CI 1.28–2.17), diabetes (SMR 3.19, 95% CI 2.19–4.49), hypertension (SMR 1.05, 95% CI 0.7–1.51), and overweight/obesity (SMR 1.18, 95% CI 0.96–1.43) compared with the general population; a high CVD risk in men was established; however, specific evaluation of CVD risk in men with HH was no analyzed.

Patients with hypogonadism should be treated to prevent or ameliorate cardiovascular disease, mainly in young men. In hypergonadotropic hypogonadism, the positive effect of TTh on cardiovascular disease and urologic symptoms has been corroborated; however, in young patients with NFPMA and HH, the information is scarce. In our study, we evaluated the effect of TTh on cardiovascular risk, metabolic profile, LUTS, and sexual function in this specific population [7, 8].

Respect to cardiovascular risk, an increase of 10-year risk of fatal CVD risk was not evidenced. The stratification of CVD risk (according to Globorisk score) evidences a low percentage of patients with high CVD risk before TTh, without modification after the treatment. An important point is the reduction of smoking and that all patients with diabetes, hypertension, and dyslipidemia received the correspondent treatment, which could contribute to the improvement of cardiovascular outcomes.

Permpongkosol et al. evidenced that TTh is related with an improvement of metabolic parameters as waist circumference, percentage of body fat, HbA1c, TC, and LDL-C at 8 years of treatment [9].

In a meta-analysis, a relationship between type 2 diabetes and lower *T* plasma levels was demonstrated; likewise, TTh was associated with a reduction in fasting plasma glucose, HbA1c, fat mass, and triglycerides, without difference in TC, HDL-C, blood pressure, and BMI [10]. In our study, we do not evidence modifications in the rate of diabetes, hypertension, and obesity; however, we evidence statistical differences in tobacco, dyslipidemia, and MetS (Table 1).

The evaluation of LUTS and sexual function in patients with TTh is important; IIEF-5 and IPSS are useful tools for this approach. Hypogonadism is related with higher IPSS score and severe LUTS, with an improvement after TTh [7], predominantly at 6 months of its administration, without effect in BMI, residual urine volume, or prostate volume, however, with elevation of PSA [11].

LUTS are related with diminution of QoL, representing the independent parameter that most influenced the decision to start TTh [12]. There was evidence that TTh improves nocturia, sleep conditions, and QoL among men with hypogonadism [8]. In our study, we do not evidence changes in QoL.

A meta-analysis reported that TTh was associated with an increase in sexual desire or libido and improvement of erectile function and sexual satisfaction, without effect on energy, mood, or LUTS [13]. In our series, TTh was associated with a lower IPSS score and a minor rate of intermittency and episodes of nocturia.

A meta-analysis that evaluated the effects of TTh evidenced an elevation of PSA, hematocrit, and bone mineral density, without major adverse cardiovascular events or prostate cancer, concluding that long-term TTh was safe, tolerable, and effective on metabolic outcomes [9]. In our study, the adverse TTh reactions were evidenced in a minority of patients, with a minor severity. Several studies report that TTh does not increase prostate cancer rates or LUTS/BPH progression, supporting the prostate safety [14]. In this study, PSA delta >1.4 ng/mL was detected in one patient, with the evidence of malign neoplasm, with a multidisciplinary approach.

The limitations of our study include its retrospective nature and the low statistical power to establish the association between TTh and cardiovascular outcomes. Future researches are necessary to determinate CVD risk at long-term TTh in this specific population.

## 5. Conclusions

TTh had a positive effect on cardiovascular risk, metabolic profile, LUTS, and sexual function in young men with nonfunctional pituitary macroadenoma and hypogonadotropic hypogonadism, and it is relatively safe in the prostatic context. The evaluation of cardiovascular and reproductive function is fundamental during TTh.

## Figures and Tables

**Table 1:** Biochemical characteristics of patients with NFPMA, HH, and TTh (*n*=118).

	Before TTh	At the last checkup with TTh	*p* value
Diabetes (%)	17	19	0.84
Hypertension (%)	22	27	0.85
Tobacco (%)	11	5	**0.05**
Dyslipidemia (%)	40	52	**0.03**
Obesity (%)	30	32	0.40
Metabolic syndrome (%)	25	34	**0.05**
Glucose (mg/dL), median (IQR)	94 (88–103)	92 (86–102)	0.40
HbA1c (%), mean ± SD	6.3 ± 1.9	6.0 ± 1.3	0.10
Systolic blood pressure (mmHg), mean ± SD	115 ± 12	116 ± 14	0.60
Diastolic blood pressure (mmHg), mean ± SD	72 ± 10	72 ± 9	0.56
Total cholesterol (mg/dL), median (IQR)	191 (174–223)	186 (166–213)	0.07
HDL-C (mg/dL), median (IQR)	39 (32–47)	40 (34–46)	0.13
LDL-C (mg/dL), median (IQR)	98 (76–156)	102 (90–122)	0.37
Triglycerides (mg/dL), median (IQR)	200 (153–294)	174 (134–233)	**0.03**
Uric acid, mean ± SD	6.1 ± 1.4	6.4 ± 1.4	0.10
Globorisk score	7 (4–10)	7 (5–11)	0.60
Testosterone	195 (101–259)	574 (423–774)	**0.01**

**Table 2:** The International Index of Erectile Function (IIEF-5) Questionnaire (*n*=118).

	Total	Confidence to get and keep an erection	Penetration quality	Erection after penetration	Erection to completion of intercourse	Satisfaction of sexual intercourse
Before TTh	15.5 ± 6.5	1.7 ± 1.2	2.0 ± 1.5	1.8 ± 1.2	1.8 ± 1.2	1.8 ± 1.3
At the last checkup with TTh	17.8 ± 5.3	1.9 ± 1.0	2.6 ± 1.3	2.5 ± 1.6	2.4 ± 1.3	2.5 ± 1.5
*p* value	0.11	0.36	**0.05**	**0.02**	**0.03**	**0.01**

**Table 3:** International prostate symptom score (*n*=118).

	Before TTh	At the last checkup with TTh	*p*
Total	7 (4–12)	6 (2–10)	0.30
	8.5 ± 6.5	7.41 ± 6.33	0.38
Incomplete bladder emptying	1 (0–2)	1 (0–1)	0.48
	1.2 ± 1.3	1.13 ± 1.43	0.70
Urinary frequency	1 (1–2)	1 (0–2)	0.71
	1.4 ± 1.25	1.43 ± 1.49	0.90
Urinary intermittency	1 (0–2)	0 (0–1)	**0.03**
	1.2 ± 1.5	0.6 ± 1.3	0.06
Urgency	1 (0–1)	0 (0–1)	0.22
	1.03 ± 1.17	0.9 ± 1.42	0.60
Weak stream	1 (0–1.5)	1 (0–2)	0.70
	1.14 ± 1.35	1.23 ± 1.61	0.90
Straining	0 (0–1)	0 (0–1)	0.60
	0.6 ± 1.2	0.58 ± 1.16	0.70
Nocturia	1 (1–2.5)	1 (1–2)	**0.04**
	1.78 ± 1.13	1.38 ± 1.23	0.13
*Quality of life*	2 (1–3)	2 (1–2)	0.60
	1.92 ± 1.27	1.87 ± 1.35	0.70

**Table 4:** Adverse effects of testosterone therapy (*n*=118).

	Before TTh	At the last checkup with TTh	*p*value
Prostate-specific antigen (ng/mL), median (IQR)	0.5 (0.3–1.03)	0.74 (0.5–1.3)	**0.02**
Hematocrit (%), mean ± SD	44.3 ± 4.5	47.6 ± 4.7	**0.001**
Hemoglobin (g/dL), mean ± SD	14.9 ± 1.5	15.9 ± 1.5	**0.001**
Aspartate aminotransferase, AST (U/L), mean ± SD	25 (21–34)	23 (19–29)	0.09
Alanine aminotransferase, ALT (U/L), mean ± SD	30 (22–42)	24 (19–34)	0.30

## Data Availability

All data generated or analyzed during this study are included in this published article. The database generated during the current study is available with the corresponding author on reasonable request.

## Abbreviations

NFPMA: Nonfunctional pituitary macroadenoma
HH: Hypogonadotropic hypogonadism
T: Testosterone
AD: Androgen deficiency
TTh: Testosterone therapy
LUTS: Low urinary tract symptoms
BPH: Benign prostatic hyperplasia
CVD: Cardiovascular disease
QoL: Quality of life
PSA: Prostate-specific antigen
IIEF-5: International index of erectile function
IPSS: International prostate symptoms score
BMI: Body mass index
MetS: Metabolic syndrome.
